# Supplementary material for: Exploring Anatomo-Morphometric Characteristics of Infrapatellar, Suprapatellar Fat Pad, and Knee Ligaments in Osteoarthritis Compared to Post-Traumatic Lesions
Source: Biomedicines. 2022 Jun 9;10(6):1369. doi: 10.3390/biomedicines10061369 (PMC9220326; doi:10.3390/biomedicines10061369)
Supplement: Supplementary file 1 [file biomedicines-10-01369-s001.zip › biomedicines-1717041-supplementary.pdf]

Supplementary file

Table S1. ACL and PCL MR morphometric characteristics in patients with meniscal tear, ACLR and end-stage OA.

	Meniscal tear	ACLR	End-stage OA	P
**ACL volume (mm³)**	413.0 (511.7-338.1)		453.7 (655.7-264.3)	p=0.865
**ACL surface (mm²)**	529.8 (631.7-469.4)		551.1 (695.5-408.0)	p=0.830
**ACL length (mm)**	34.4 (39.0-32.6)		33.8 (37.4-29.9))	p=0.296
**PCL volume (mm³)**	928.2 (1285.1-775.4)	924.2 (1144.1-757.7)	973.1 (1041.7-782.2)	p=0.860
**PCL surface (mm²)**	848.5 (988.9-713.1)	823.1 (954.9-745.6)	838.4 (906.0-763.3)	p=0.856
**PCL length (mm)**	39.3 (43.4-35.7)	41.5 (45.2-38.5)	39.1 (43.9-36.3)	p=0.117

ACL = anterior cruciate ligament, PCL = posterior cruciate ligament, MR = magnetic resonance; ACLR = anterior cruciate ligament rupture; OA = osteoarthritis.
Data are expressed as median (Interquartile range).

Table S2. Multivariate analysis of IFP volume.

	Estimate	Standard error	t-value	p-value
**Intercept**	25745.25	4273.17	6.025	p<0.001
**Age**	30.03	53.91	0.557	p=0.5790
**Sex**	-10366.45	1272.36	-8.147	p<0.001
**BMI**	242.10	129.95	1.863	p=0.0660
**ACL**	4002.23	1520.55	2.632	p=0.0101
**OA**	-4452.37	1854.01	-2.401	p=0.0186

IFP = infrapatellar fat pad; BMI = body mass index; ACL = anterior cruciate ligament; OA = Osteoarthritis.

Table S3. Multivariate analysis of IFP surface.

	Estimate	Standard error	t-value	p-value
**Intercept**	6118.64	886.72	6.90	p<0.0001
**Age**	6.73	11.19	0.60	p=0.549
**Sex**	-1869.96	264.03	-7.08	p<0.0001
**BMI**	60.17	26.97	2.23	p=0.028
**ACL**	1028.56	315.60	3.26	p=0.002
**OA**	-783.40	384.73	-2.04	p=0.045

IFP = infrapatellar fat pad; BMI = body mass index; ACL = anterior cruciate ligament; OA = Osteoarthritis.

Table S4. Multivariate analysis of IFP depth*.

	Estimate	Standard error	t-value	p-value
**Intercept**	903.01	205.45	4.39	p<0.001
**Age**	2.13	2.59	0.82	p=0.413
**Sex**	-298.13	61.33	-4.86	p<0.001
**BMI**	0.72	6.20	0.12	p=0.910
**ACL**	88.62	74.72	1.19	p=0.239
**OA**	-266.51	88.71	-3.00	p=0.004

IFP = infrapatellar fat pad; BMI = body mass index; ACL = anterior cruciate ligament; OA = Osteoarthritis.
*the variable did not follow a normal distribution, thus the square of variable was analyzed.

Table S5. Multivariate analysis of IFP Femoral arch length.

	Estimate	Standard error	t-value	p-value
**Intercept**	903.01	4.76	6.42	p<0.0001
**Age**	0.09	0.06	1.47	p=0.145
**Sex**	-5.75	1.42	-4.04	p<0.0001
**BMI**	-0.09	0.14	-0.62	p=0.540
**ACL**	1.29	1.73	0.75	p=0.457
**OA**	-3.93	2.06	-1.91	p=0.059

IFP = infrapatellar fat pad; BMI = body mass index; ACL = anterior cruciate ligament; OA = Osteoarthritis.

Table S6. Multivariate analysis of IFP tibial arch length*.

	Estimate	Standard error	t-value	p-value
**Intercept**	1103.05	248.34	4.44	p<0.0001
**Age**	3.90	3.13	1.24	p=0.217
**Sex**	-270.23	74.14	-3.64	p<0.001
**BMI**	-3.72	7.49	-0.50	p=0.621
**ACL**	-86.50	90.32	-0.96	p=0.341
**OA**	-366.70	107.22	-3.42	p<0.001

IFP = infrapatellar fat pad; BMI = body mass index; ACL = anterior cruciate ligament; OA = Osteoarthritis.

*the variable did not follow a normal distribution, thus the square of the variable was analyzed.

Table S7. Multivariate analysis of SFP volume*.

	Estimate	Standard error	t-value	p-value
**Intercept**	6.24	0.34	18.28	p<0.0001
**Age**	0.005	0.005	1.18	p=0.243
**Sex**	-0.22	0.10	-2.28	p=0.025
**BMI**	0.02	0.01	2.28	p=0.025
**ACL**	0.56	0.12	4.69	p<0.0001
**OA**	-0.15	0.14	-1.07	p=0.288

SFP = suprapatellar fat pad; BMI = body mass index; ACL = anterior cruciate ligament; OA = Osteoarthritis.
* the variable did not follow a normal distribution, thus the logarithm of the variable was analyzed.

Table S8. Multivariate analysis of SFP surface*.

	Estimate	Standard error	t-value	p-value
**Intercept**	6.23	0.26	23.82	p<0.0001
**Age**	0.003	0.003	1.10	p=0.279
**Sex**	-0.18	0.07	-2.40	p=0.019
**BMI**	0.02	0.01	2.14	p=0.035
**ACL**	0.33	0.09	3.64	p<0.001
**OA**	-0.14	0.11	-1.30	p=0.196

SFP = suprapatellar fat pad; BMI = body mass index; ACL = anterior cruciate ligament; OA = Osteoarthritis.
* the variable did not follow a normal distribution, thus the logarithm of the variable was analyzed.

Table S9. Multivariate analysis of IFP hypointense signal.

	Estimate	Standard error	z-value	p-value
**0|1**	1.184	1.682	0.704	0.481
**1|2**	3.122	1.719	1.816	0.069
**2|3**	4.845	1.763	2.748	0.006
**Age**	0.009	0.020	0.44	p=0.662
**Sex**	-1.35	0.53	-2.54	p=0.011
**BMI**	0.03	0.05	0.54	p=0.590
**ACL**	0.72	0.58	1.21	p=0.225
**OA**	3.71	0.78	4.72	p<0.0001

IFP = infrapatellar fat pad; BMI = body mass index; ACL = anterior cruciate ligament; OA = Osteoarthritis.

Table S10. Multivariate analysis of SFP hypointense signal.

	Estimate	Standard error	z-value	p-value
**0|1**	-2.602	1.585	0.704	0.101
**1|2**	-0.621	1.557	1.816	0.690
**Age**	-0.019	0.019	-0.996	p=0.319
**Sex**	-0.24	0.47	-0.51	p=0.609
**BMI**	-0.01	0.05	-0.21	p=0.832
**ACL**	-1.83	0.58	-3.12	p=0.002
**OA**	0.36	0.69	0.53	p=0.598

SFP = suprapatellar fat pad; BMI = body mass index; ACL = anterior cruciate ligament; OA = Osteoarthritis.

Table S11. Multivariate analysis of PL depth.

	Estimate	Standard error	t-value	p-value
**Intercept**	5.33	0.49	10.87	p<0.0001
**Age**	-0.019	0.006	-1.76	p=0.082
**Sex**	-0.50	0.15	-3.43	p<0.001
**BMI**	-0.02	0.01	-1.21	p=0.229
**ACL**	-0.26	0.18	-1.50	p=0.137
**OA**	0.09	0.21	0.44	p=0.659

PL = patellar ligament; BMI = body mass index; ACL = anterior cruciate ligament; OA = Osteoarthritis.
